# Risk Factors for Endophthalmitis and Retinal Detachment with Retained Intraocular Foreign Bodies

**DOI:** 10.1155/2012/758526

**Published:** 2012-04-26

**Authors:** D. Wilkin Parke, Avinash Pathengay, Harry W. Flynn, Thomas Albini, Stephen G. Schwartz

**Affiliations:** Department of Ophthalmology, Bascom Palmer Eye Institute, Miller School of Medicine, University of Miami, Miami, FL 33136, USA

## Abstract

*Purpose*. To analyze risk factors for endophthalmitis and retinal detachment (RD) in patients with retained intraocular foreign bodies (IOFBs). *Design*. A retrospective, interventional, consecutive case series. *Participants*. All patients treated at Bascom Palmer Eye Institute for traumatic IOFBs between 1999 and 2008. *Methods*. Analysis of visual outcome, mechanism of injury, management, and postoperative course. *Results*. 108 eyes with IOFBs were identified. Endophthalmitis occurred in 7 eyes (6.4%) at presentation, and risk was higher with vegetable matter exposure (*P* = 0.003). All eyes with posterior segment IOFBs received intravitreal antibiotics and there were no cases of endophthalmitis after initial management. RD was identified in 6 of 108 eyes (5.5%) at presentation. Risk factors were entry more than 5 mm behind the limbus (*P* < 0.001) and posterior segment IOFB (*P* = 0.028). Postoperative RD occurred in 11 of 102 eyes (10.7%). Risk factors for postoperative RD were preoperative endophthalmitis (*P* = 0.001), posterior segment IOFB (*P* = 0.008), and retinal impact sites (*P* = 0.028). *Conclusions*. Risk factors for endophthalmitis included vegetable matter exposure and delay to initial management. Risk factors for RD were posterior entry site, posterior segment IOFB, endophthalmitis, and retinal impact sites. No eyes developed endophthalmitis after presentation.

## 1. Introduction

Retained intraocular foreign bodies (IOFBs) are potential sources of severe visual loss. IOFBs have been estimated to occur in 10–41% of open-globe injuries [1–4]. Severe visual loss may be associated with the original injury, secondary endophthalmitis, rhegmatogenous retinal detachment (RD), or a variety of long-term complications including glaucoma, cataract, inflammation, or foreign body toxicity. Traumatic endophthalmitis and RD are of particular concern given their tendency towards rapid progression resulting in severe visual loss [3, 5–7]. Endophthalmitis has been reported in 2–30% of open-globe injuries with retained IOFBs [8–10], and an IOFB may be present in 43% of eyes with traumatic endophthalmitis [11]. RD has been reported in 6–30% of eyes with IOFBs [6, 12, 13].

 Although the ideal timing of IOFB removal is early, this may not always be clinically feasible. The current study investigates risk factors for endophthalmitis and RD in patients with retained IOFBs, employing a retrospective cohort of patients with a history of IOFB treated at a single ocular trauma center over a ten-year period.

## 2. Patients and Methods

The study was performed with the approval of the University of Miami Institutional Review Board and in accordance with the U.S. Health Insurance Portability and Accountability Act and the Declaration of Helsinki. A database search was performed via diagnostic and procedural codes of all patients presenting to Bascom Palmer Eye Institute with open-globe injuries and associated traumatic IOFBs between January 1st, 1999 and December 31st, 2008. Surgically induced retained IOFBs such as intraocular lenses were excluded from the current study. Intraorbital, extraocular IOFBs were excluded. IOFBs were defined as intraocular if all or part of the foreign material was within the intraocular space at time of presentation.

 Medical records were reviewed for data regarding patient demographics, mechanism and details of injury, visual acuity (VA) and examination results at presentation and postoperatively, any diagnostic imaging, and surgery. The sensitivity of the imaging modalities for recognition of IOFBs was evaluated by identifying the IOFBs found at time of surgery and searching for correct detection of these IOFBs on preoperative imaging reports. Surgical details included timing, type, and number of procedures, as well as the intraoperative findings or administration of pharmaceutical agents. The intervals between injury, presentation, and treatment were noted. Postoperative followup was included extending up to two years after injury when available. Factors directly contributing to decreased visual acuity were determined when possible from the retrospective review of the medical records.

 The Ocular Trauma Classification Group system for zone of entry was applied, with corneal entry referred to as zone one, entry within 5 mm posterior to the limbus as zone two, and anything posterior to zone two as zone three [14]. In cases of large wounds crossing multiple zones, the zone involved in the most posterior aspect of the entry wound was used for this classification.

 Visual acuity was converted from Snellen to logMAR scale for the purpose of calculating mean values and converted back to Snellen score for data presentation. LogMAR values of 2, 3, 3.5, and 4 were assigned to visual acuity scores of count fingers at 1 foot (CF 1′), hand motion (HM), light perception (LP), and no light perception (NLP), respectively, as have been utilized previously at the study institution [15]. Statistical analysis was performed with the two-tailed student *t*-test for continuous variables and the Fischer test for categorical data. *P* value less than 0.05 was considered significant.

## 3. Results

A total of 108 eyes in 107 patients had IOFBs and underwent treatment; one patient had bilateral IOFBs. Mean age among the 107 patients was 31.4 years (range 7–66), with 104 (97%) male. The mean time from injury to presentation was 3.5 days with a range of 2 hours to 248 days. Excluding one extreme value of 248 days, the mean time was 1.8 days with range 2 hours to 18 days. Mean followup was 54.4 days, with range 8 days to 2 years.

 Mean best corrected visual acuity (BCVA) for all eyes was 20/600 (logMAR 1.48, range 20/20 to NLP) preoperatively and 20/210 (logMAR 1.03, range 20/20 to NLP) postoperatively (*P* = 0.038). Foreign body location within the eye (as distinguished from zone of entry) was divided into anterior segment, of which an intralenticular subset was identified, posterior segment (posterior to posterior lens capsule), and traversing (long IOFBs protruding from the eye and extending into the posterior segment). Anterior segment IOFBs were identified in 41 eyes (38.0%), of which 29 eyes (70.7%) were anterior to the lens, with a mean preoperative VA of 20/80 (logMAR 0.60), and postoperative last corrected VA of 20/63 (logMAR 0.50) (*P* = 0.84). Twelve of the 41 anterior segment IOFBs (29.2%) were intralenticular, with preoperative VA 20/370 (logMAR 1.275) and last corrected VA 20/36 (logMAR 0.26) (*P* = 0.006). Fifty-seven eyes (53%) had posterior segment IOFBs, with preoperative VA 20/2200 (logMAR 2.04) and last corrected VA 20/740 (logMAR 1.57) (*P* = 0.122). Ten IOFBs (9%) were traversing, with preoperative VA 20/520 (logMAR 1.42) and last corrected VA 20/350 (logMAR 1.25) (*P* = 0.82). Six of 108 eyes (6%) underwent enucleation, 2 as a primary surgery and 4 as a secondary procedure.

 Analysis of the IOFB type was available for 67 of 108 eyes (62%) (Table 1). The majority of foreign bodies were magnetic. Fifty six of 67 samples (84%) were described on pathology report as being magnetic, versus 2 (3%) that were nonmagnetic metal, and 9 (13%) that were nonmetallic. The mean largest dimension of the IOFB on pathology was 5.4 mm (range 0.5–17 mm). Sixty eight of 108 eyes (63%) had IOFBs visible on clinical examination alone at presentation, and 12 (11%) were protruding through the presumed entry site at time of examination. The entry site was identifiable preoperatively in 93 eyes (86%), intraoperatively in an additional 4 eyes (4%), and could not be determined in 11 eyes (10%). Fifty two eyes (48%) had zone one, 38 eyes (35%) had zone two, and 7 eyes (6%) had zone three entry. Zone three entry was associated with a higher rate of enucleation (4 of 7 eyes, *P* < 0.001) and poor final visual acuity (<5/200 in the two nonenucleated eyes).

 Time interval to surgical removal of retained IOFBs was measured from presentation and from reported injury. Two of the 108 eyes (2%) underwent primary enucleation. One hundred and three of the remaining 106 eyes (97%) underwent removal of IOFB within 24 hours of presentation. This stands in distinction from the interval from injury to IOFB removal, which was much more variable. Mean time from injury to IOFB removal was 3.1 days, excluding two outliers which were operated 137 and 276 days after injury, both of which had occult posterior segment IOFBs with moderate inflammation. Anterior segment IOFBs were removed a mean of 3.3 days after injury, intralenticular IOFBs a mean of 14.4 days after injury, posterior segment IOFBs 3.1 days after injury, and traversing IOFBs 1.1 days after injury. The 3 eyes (3%) in which IOFB removal was delayed more than 24 hours after presentation underwent IOFB removal at 7, 28, and 137 days after presentation. Removal was delayed 7 days in the first patient because the patient left against medical advice prior to the first scheduled surgery and then returned 5 days later. Final BCVA in this patient was 20/800. The second patient presented 248 days after injury with an intralenticular IOFB and was observed for 28 days prior to removal, and final BCVA was 20/20. The third patient had an intralenticular IOFB that was observed for 137 days until visually significant cataract developed, and final BCVA was 20/20.

 Endophthalmitis occurred in 7 of 108 eyes (6.5%), all clinically evident at time of presentation before initial surgery (Table 2). All 7 were culture positive. No eyes developed endophthalmitis after IOFB removal during followup. Five of the 7 eyes (71%) with endophthalmitis upon presentation had posterior segment IOFBs, and 2 of 7 (29%) had traversing IOFBs. Both of the traversing IOFBs causing endophthalmitis were magnetic wires extending through anterior segment, lens, vitreous, and into the retina. Preoperative VA in the eyes with endophthalmitis was HM in 6 eyes and NLP in 1 eye (mean logMAR 3.14). Postoperative last corrected VA in the endophthalmitis eyes was 10/200 in 1 eye, 5/200 in 3 eyes, 1/200 in 1 eye, HM in 1 eye, and NLP in 1 eye (the same eye that was NLP preoperatively). The mean logMAR visual acuity on last examination was 2.16. The eye that was NLP after initial repair underwent secondary enucleation. Risk factors for endophthalmitis included a mechanism of injury related to vegetable matter exposure from yard work (3 of 7 eyes, *P* = 0.003), nonmetallic IOFBs (3 of 7 eyes, *P* = 0.081), and time interval from injury to presentation (2.7 days versus 1.8 days in eyes without infection, *P* = 0.19).

 All patients with IOFBs involving the posterior segment or posterior capsular violation received prophylactic intravitreal antibiotics (vancomycin 1 mg/0.1 cc and ceftazidime 2.25 mg/0.1 cc) at time of primary surgery. Use of prophylactic intravitreal or intracameral antibiotics with anterior segment IOFBs occurred in 35 of 41 eyes (85.4%). Systemic antibiotic usage varied over the ten-year study period, but generally included a single dose of an intravenous fluoroquinolone. No patients developed direct evidence of siderosis or toxicity due to the foreign body substance during the followup period.

 RD occurred in 18 eyes (16.7%). The presence of RD before initial surgery and postoperatively was analyzed separately. The retina was visible on initial fundus examination in 66 of 108 eyes (61.1%), not visible in 35 eyes (32.4%), and not noted in the record in 7 eyes. Six out of 108 eyes (5.6%) had an RD preoperatively. Risk factors for RD at presentation were zone three entry site and posterior segment IOFB. Six of 6 eyes with RD at presentation had Zone Two or Three injury, compared to 39 of the other 102 eyes (*P* = 0.004). The mean value for zone of entry was 2.7 in eyes with preoperative RDs, as opposed to 1.3 in the eyes not presenting with RD (*P* < 0.001). All 6 eyes with RD involved posterior segment IOFBs, whereas only 53% of all IOFBs were posterior segment (*P* = 0.028). One of the 6 eyes with preoperative RD also had endophthalmitis. Final BCVA in this eye was 5/200. Preoperative RD was not statistically associated with size of entry wound or IOFB material. All 6 eyes underwent pars plana vitrectomy, IOFB removal, endolaser, and long acting tamponade (3 with C3F8 gas, 3 with silicone oil). Three eyes also underwent encircling scleral buckles. Five out of the 6 eyes had postoperative recurrent RDs and required a second vitrectomy with or without sclera buckle. Mean preoperative VA was 20/4000 (range 2/200 to HM, mean logMAR 2.33), and postoperative last corrected VA was 20/3100 (range 10/200 to LP, mean logMAR 2.20).

 Development of postoperative RD was noted in 11 of 102 eyes (11%) with no preoperative RD. Risk factors for postoperative RD were posterior segment IOFB (*P* = 0.008), preoperative endophthalmitis (*P* = 0.001), and one or more retinal impact sites visualized intraoperatively (*P* = 0.028). Ten of 11 eyes involved posterior segment IOFBs, 1 of 11 eyes involved a nail that traversed the eye and rested on the retina, and 4 of 11 eyes had preoperative endophthalmitis. Seven of 11 eyes had IOFB impact damage on the retina with hemorrhage or pigmentary change visualized intraoperatively. In comparison, only 12 of the other 46 eyes with posterior segment IOFBs and 4 of 10 eyes with traversing IOFBs had visible retinal impact sites. Initial surgical approach in all 11 eyes involved pars plana vitrectomy with IOFB removal. Scleral buckling was performed in 3 eyes, and lensectomy in 6 eyes. Second surgery for repair of RD was performed in 8 of 11 eyes. One of 11 eyes required enucleation, and 2 of 11 were observed with RDs and low visual potential.

 Diagnostic imaging was employed preoperatively in 77 of 108 eyes (73%). 43 of 108 eyes (41%) underwent computerized tomography (CT). All CT scans included head and thin (1 mm) sections through the orbits with axial and coronal planes. Echography was performed in 45 of 108 eyes (42%), all done by experienced echographic technicians. The sensitivity of CT was 100% when it was obtained, detecting 12 of 12 anterior segment, 26 of 26 posterior segment, and 5 of 5 traversing IOFBs. Echography was 98% sensitive when it was obtained, detecting 15 of 16 anterior segment, 26 of 26 posterior segment, and 3 of 3 traversing IOFBs, respectively. One intralenticular anterior segment IOFB was not detected by echography. Echography reports indicated vitreous hemorrhage in 4 of 45 eyes (9%), RD in 1 of 45 eyes (2%), and opacities consistent with endophthalmitis in 1 of 45 eyes (2%).

## 4. Discussion

The presenting and postoperative visual acuities in this study are comparable to those of prior reports [3, 6, 8, 10, 16]. Prior studies indicate that presenting BCVA and IOFB location are strongly correlated with final BCVA; IOFBs that penetrate the posterior segment have a poor visual prognosis, probably secondary to mechanical damage to the retina and optic nerve, as well as higher risk of endophthalmitis and RD [17, 18]. Thus, the eyes in this study were analyzed in subgroups based on IOFB location. As previously reported, IOFBs present anterior to or within the lens had a significantly better visual acuity both on presentation and after treatment than those in the posterior segment. Intralenticular foreign bodies can be observed for variable periods of time, as their damage is frequently limited to cataract formation. The “traversing” IOFBs included larger foreign objects such as nails, wires, and fish hooks that penetrated through the anterior segment into the posterior segment but were partially protruding from the eye. This group resembled the “posterior segment” category in involving the vitreous space and often the retina but behaved differently in providing a potential path of entry from the extraocular to the intraocular environment, and thus increasing the potential for endophthalmitis. Otherwise, these eyes had better visual acuity than other eyes with IOFBs restricted to the posterior segment, possibly because they involved lower velocity foreign bodies and were frequently thin lengths of metal that could be removed with relatively less trauma. Visual outcome overall was greatly affected by the presence or absence of endophthalmitis and RD, both of which significantly decreased the last corrected visual acuity. Other previously described risk factors for poor final visual acuity including size of IOFB and time to IOFB removal were not found to be significant [16].

 Endophthalmitis has been reported in 3–11% of open-globe injuries, and 3–17% of injuries with IOFBs [17–21]. Previous reports associate a delay in removal of the IOFB with increased risk of endophthalmitis [22]. Colyer et al. reported 79 cases of endophthalmitis in eyes with combat-related IOFBs, all of which received rapid wound closure and systemic antibiotics but delayed removal of the IOFB, suggesting that at least in the setting of combat, timely antibiotic administration may be more important than immediate IOFB removal [8]. Six patients (6%) with IOFB in this study presented with endophthalmitis. Endophthalmitis eyes trended towards a longer time interval between injury and presentation (mean 2.9 versus 1.3 days), but the difference was not statistically significant. Posterior segment IOFB and yard or soil-related injuries involving vegetable matter exposure were risk factors for endophthalmitis, both of which have been previously identified [23]. None of the patients in this study developed endophthalmitis after presentation or surgery. A previous study reported the rate of postoperative endophthalmitis in open-globe injuries at around 3% [24]. Eyes with posterior segment and traversing IOFBs—those at highest risk for endophthalmitis—received prophylactic broad-spectrum intravitreal antibiotics at time of surgery in the current study, perhaps contributing to the lack of postoperative infection. Prophylactic intravitreal antibiotics for open-globe injuries with or without IOFBs remain controversial [13, 20, 25, 26].

 RD has been reported to occur in up to 30% of open-globe injuries and 6–36% of those with posterior segment IOFBs [6, 12, 13, 27]. IOFBs are frequently associated with hyphema, cataract, vitreous hemorrhage, and other media opacities that limit retinal exam and make it difficult to identify RDs preoperatively [24]. The current study distinguished preoperative RDs which were visible on exam, echography, or in the operating room upon start of surgery from those that developed after primary repair of the open-globe injury. Preoperative RDs occurred in 6 eyes (6%) and were associated with posterior segment IOFBs, endophthalmitis, and posterior entry wounds. Postoperative RDs were likewise associated with posterior segment IOFBs and endophthalmitis, but additionally at risk in eyes with IOFB impact sites on the retina. These areas may predispose to retinal breaks and proliferative vitreoretinopathy that manifest after vitrectomy. Wound length and IOFB size have been previously described as risk factors for RD but were not correlated in this study [18].

 CT has become the imaging modality of choice in many areas for screening of open-globe injuries and detection of IOFBs. Most practice locations have access to nearby CT machines, the scan is reasonably quick and user-independent, and newer orbital imaging protocols involve 1-2 mm sections that accurately detect open-globe injuries and IOFBs. Echography is less commonly used in the setting of open-globe injuries because it is a contact scan with risk of external pressure that could gape wounds or extrude intraocular contents, it requires more user experience to perform, and it does not effectively image adjacent areas like the posterior orbit, sinuses, and intracranial space [28]. Echography is potentially advantageous for its immediate availability in many ophthalmic clinics and its superior imaging of intraocular pathology such as vitreous hemorrhages, RDs, and endophthalmitis [29]. In the current study, 43% of eyes underwent CT, and 45% underwent echography. This is probably not representative of a typical practice setting, as this study was performed in an institution with an experienced echographic department. Both modalities were highly sensitive when utilized, with CT detecting 100% of IOFBs and echography detecting 98% in those patients. Thirty seven percent of IOFBs in the current study were not detected on initial clinical exam. This together with the high sensitivity of these two imaging techniques emphasizes the need for clinical suspicion and appropriate ancillary testing in cases at risk for IOFBs.

 The current study is limited by its retrospective methodology, the variability in the included pathology, and the limited followup in many patients. The number of endophthalmitis cases was relatively small. Multiple surgeons were involved, and there was a spectrum of operative approaches, particularly regarding removal of the IOFB, endotamponade selection, and lens management.

 In summary, injury in the setting of soil or vegetable matter exposure, nonmetallic IOFBs, and longer time to presentation are risk factors for endophthalmitis in patients with IOFBs. Prophylactic intravitreal antibiotics at time of IOFB removal may lower this risk. RD is associated with posterior segment IOFBs, posterior entry wounds, endophthalmitis, and retinal impact sites.

## Figures and Tables

**Table 1:** IOFB characteristics and visual acuity by location.

	No. of IOFBs (%)	No. of magnetic (%)	Mean size (mm)	Mean zone of entry	Initial VA	Last corrected VA
All locations	108	56/67 (84)	3.93	1.27	20/600	20/210
Anterior segment	29 (27%)	10/16 (63)	3.45	1.2	20/80	20/63
Intralenticular	12 (11%)	6/7 (86)	1.67	1.00	20/370	20/36
Posterior segment	57 (53%)	30/34 (88)	3.78	1.35	20/2200	20/740
Traversing	10 (9%)	10/10 (100)	14.5	2.00	20/520	20/350

**Table 2:** Endophthalmitis in eyes with IOFBs: demographics, organisms, and outcomes.

Case no.	Age	Mechanism of injury	Time from injury to presentation (days)	Time from injury to IOFB removal (days)	Culture result	IOFB	Initial corrected VA	Last corrected VA	Postoperative course
1	55	Mowing lawn	4	4	*Enterococcus* sp.	Magnetic wire 16 mm	HM	20/400	No further surgery
2	42	Hammering metal	6	6	*S. aureus*	Magnetic 1 mm	HM	1/200	No further surgery
3	31	Hammering metal	1	1	*Bacillus* sp., *S. epidermidis *	Magnetic 2 mm	HM	20/800	Ret. detachment, PPV/MP/SOX
4	50	Using weed eater	5	8	*Bacillus* sp.	Nonmetal 1 mm	HM	20/800	Ret. detachment, PPV/SB/MP/SOI
5	32	Hammering metal	3	5	*Moraxella* sp.	Nonmetal 1 mm	HM	HM	Ret. Detachment, PPV/SB/SOI
6	39	Mowing lawn	0.25 (6 hours)	0.5 (10 hours)	*P. acnes*	Magnetic wire 14 mm	HM	2/200	No further surgery
7	41	Hammering metal	1	1	*Bacillus* sp.	Nonmetal 2 mm	NLP	NLP	Enucleation
